# Pregnancy Outcomes After in Utero Exposure to Immune Checkpoint Inhibitors

**DOI:** 10.3390/curroncol33060318

**Published:** 2026-05-28

**Authors:** Morgan Bou Zerdan, Bruna Kfoury, Eliane Aoun, Sarah Diane Hmaidan, Roni Nitecki Wilke, Jeffrey A. How, Terri L. Woodard, Pamela T. Soliman, Laurie J. McKenzie

**Affiliations:** 1 Department of Gynecologic Oncology and Reproductive Medicine, The University of Texas MD Anderson Cancer Center, Houston, TX 77030, USApsoliman@mdanderson.org (P.T.S.); ljmckenzie@mdanderson.org (L.J.M.); 2 School of Pharmacy, Lebanese American University, Beirut P.O. Box 13-5053, Lebanon; bruna.kfoury@lau.edu; 3 Department of Obstetrics and Gynecology, Baylor College of Medicine, Houston, TX 77030, USA; sarah.hmaidan@bcm.edu

**Keywords:** immune checkpoint inhibitors, pregnancy exposure, melanoma, pregnancy outcomes, fetal toxicity, maternal-fetal medicine

## Abstract

Importance: Immune checkpoint inhibitors (ICIs) have transformed the management of cancers affecting reproductive-age patients, yet their impact on pregnancy outcomes remains incompletely understood. We describe two cases of maternal and fetal outcomes associated with ICI exposure during pregnancy and present a comprehensive literature review. Methods: A retrospective chart review was conducted at MD Anderson Cancer Center (1 January 2015 to 31 December 2024) to identify patients exposed to ICIs during pregnancy. Clinical data including cancer type, treatment timing, pregnancy course, and maternal and neonatal outcomes were collected. A narrative literature review was also performed using PubMed to identify reported cases of ICI exposure during pregnancy. Observations: Two patients were identified at our institution, both treated with ICIs for advanced melanoma. One patient received pembrolizumab during early pregnancy, with the final dose administered five days after conception, and subsequently gave birth to a healthy term infant without complications. The second patient conceived while receiving adjuvant nivolumab and experienced a miscarriage at 13 weeks of gestation. Neither patient experienced immune-related toxicity during pregnancy, and both remained without evidence of disease at follow-up. The literature review identified 21 reported pregnancies with ICI exposure and variable outcomes. Most resulted in live births (85.7%), though preterm delivery occurred in approximately 50% of cases, often due to maternal or fetal indications. Additional reported outcomes included miscarriage, neonatal death, fetal growth restriction, preeclampsia, and rare immune-related neonatal effects. Congenital anomalies were reported in a small number of cases. Conclusions and Relevance: These findings suggest that, while many pregnancies exposed to ICIs result in live births, there may be an increased risk of adverse maternal and fetal outcomes. However, causality cannot be established due to the limited quality and quantity of available data. These findings underscore the importance of effective contraception during ICI therapy and careful multidisciplinary counseling when exposure occurs during pregnancy.

## Simple Summary

Immunotherapy has improved cancer treatment outcomes, including for younger patients who may become pregnant during therapy. However, its safety during pregnancy remains unclear. In this study, we describe two patients who were exposed to immunotherapy during pregnancy and review previously reported cases. Most pregnancies resulted in healthy live births, but there was a notable risk of complications such as miscarriage, preterm delivery, and impaired fetal growth. Rare effects on newborns were also reported. These findings highlight the importance of avoiding pregnancy during treatment and ensuring careful counseling and monitoring when exposure occurs. More research is needed to better understand the risks and guide clinical decision-making.

## 1. Introduction

Immune checkpoint inhibitors (ICIs) targeting programed cell death-1 (PD-1), its ligand PD-L1, and cytotoxic T-lymphocyte-associated antigen-4 (CTLA-4) have transformed cancer care, leading to significant survival benefits in advanced melanoma, lung cancer, renal cell carcinoma, and Hodgkin lymphoma [1]. Recently, anti-PD-1 therapy has been approved for high-risk early-stage triple-negative breast cancers [2]. As the indications for ICIs expand to include malignancies that affect reproductive-age patients, understanding the possible reproductive impacts of ICIs becomes increasingly important [3].

Safety data regarding ICIs in pregnancy remain extremely limited [4]. As in most therapeutic drug trials, pregnant patients have been excluded from clinical trials of ICIs due to concerns about fetal harm [5]. Preclinical studies show CTLA-4 blockade increases pregnancy loss, stillbirth, and infant mortality. Similarly, murine models demonstrated that PD-1/PD-L1 disruption (crucial for fetomaternal immune tolerance) can trigger fetal rejection and resorption [6,7]. This pathway is highly expressed at the placental interface to protect the semi-allogeneic fetus from maternal T-cell attack, with PD-L1 playing a central role in maintaining immune tolerance at the maternal–fetal interface [8].

The clinical experience with inadvertent ICI use during pregnancy consists of individual case reports and small series with varied outcomes [9]. Adverse maternal and fetal outcomes that have been reported in association with ICI exposure include miscarriage, preterm birth, preeclampsia, fetal growth restriction, and immune-related neonatal complications [4,10,11]. Concerns regarding ICI use in pregnancy are based on preclinical and murine data suggesting potential fetal harm, in addition to the absence of robust human data to adequately assess risk [9]. Nevertheless, healthy live births have also been reported, suggesting that adverse outcomes are not universal [12]. The lack of comprehensive data leaves clinicians with substantial uncertainty in counseling and management.

Despite increasing clinical use, the safety of immune checkpoint inhibitors during pregnancy remains highly controversial. Available clinical data, largely derived from case reports and small series, suggest that favorable outcomes are possible [13]; however, these findings are limited by small sample sizes, heterogeneity in exposure timing, and potential reporting bias [13]. In contrast, preclinical and mechanistic studies raise concerns regarding disruption of fetomaternal immune tolerance, which may lead to adverse maternal and fetal outcomes.

### Objective

We describe two cases of immune checkpoint inhibitor exposure during pregnancy and present a comprehensive review of the literature to better characterize maternal and fetal outcomes. Our aim is to synthesize current evidence, highlight potential risks, and provide clinically relevant insights to guide patient counseling and management.

Our findings suggest that, while favorable pregnancy outcomes are possible, immune checkpoint inhibitor exposure during pregnancy is associated with meaningful risks, underscoring the need for caution and multidisciplinary management.

## 2. Methods

### 2.1. Case Series

A retrospective chart review from 1 January 2015, to 31 December 2024, was conducted at MD Anderson Cancer Center to identify patients who were inadvertently exposed to ICI during pregnancy. Following Institutional Review Board approval (IRB), patient consent was waived due to the retrospective nature of the study, and two cases were identified. Data abstracted included maternal demographics, oncologic history, ICI regimen and its timing in relation to pregnancy, obstetrical complications, and fetal outcomes. Clinical details are presented in Section 3.

### 2.2. Literature Review

A narrative literature review was conducted using PubMed/MEDLINE through March 2025 to identify reports of ICI exposure during pregnancy. Search terms included PD-1, PD-L1, and CTLA-4 inhibitors (e.g., nivolumab, pembrolizumab, atezolizumab, ipilimumab) combined with pregnancy-related terms (“pregnancy, “fetal,” “maternal”, etc.). No language restrictions were applied, but only studies with available English text or translation were included. Reference lists of pertinent articles were also manually reviewed. We included publications of any design (case report, case series, registry analysis, or cohort study) reporting at least one pregnant patient treated with ICIs and maternal/fetal outcomes. Abstract-only reports were included if sufficient outcome detail was provided. Reviews and commentaries without incident cases were excluded. The extracted data aligned with our case series variables, including maternal cancer type, ICI agent(s) and therapy indication, timing of ICI exposure in pregnancy, and maternal/fetal outcomes. Given the rarity of these cases, we summarized the findings narratively and in tabular form. All published cases identified are compiled in Table 1. “Additional case details are provided in Supplementary Table S1.”

## 3. Literature Review

Our literature review yielded a growing body of reports on ICI use in pregnancy. We identified 20 publications meeting inclusion criteria, collectively reporting 21 pregnancies with ICI exposure (excluding our two new cases). These reports span from 2017—when the first case of an anti-CTLA-4 (ipilimumab) use in pregnancy was described—through early 2024, reflecting the increased recognition of such cases. Most were single-case reports, with a few larger case series and pharmacovigilance database analyses.

### 3.1. Cancer Types and ICI Agents

Most reported cases (66.6%, 14/21) involved melanoma, one of the earliest malignancies treated with ICIs. Other cancer types reported include lung cancer (non-small cell lung carcinoma; one case), renal cell carcinoma (one case), Hodgkin lymphoma (three cases), and a few cases of other malignancies (such as colon cancer and nasopharyngeal carcinoma). The mean age at diagnosis with cancer was 32.25 years (SD = 4.14), with a median age of 33 years (range: 23–39). The majority of reported pregnancies (16/21) involved exposure to ipilimumab (anti-CTLA-4) and/or nivolumab (anti–PD-1)—either as monotherapy or in combination—while the remaining five involved pembrolizumab (anti-PD-1). Timing of ICI exposure varied widely. Some women inadvertently received ICIs in early first trimester before pregnancy was recognized. While most patients discontinued immunotherapy once pregnancy was discovered, a subset of patients continued treatment through late gestation when oncologically indicated. Reported exposure timing ranged from preconception and early first-trimester exposure to continued treatment through the third trimester, with gestational exposure extending up to approximately 37 weeks in some reported cases.

### 3.2. Stage at Pregnancy

Among melanoma cases, 42.8% (6/14) of patients were diagnosed with stage IV disease during pregnancy, 14.2% (2/14) of cases were stage III, 21.4% (3/14) of cases were metastatic without formal staging, and 21.4% (3/14) of cases had no stage information. In contrast, with other malignancies, the staging information was frequently missing (85.7%, 6/7), with one case out of the seven non-melanoma cases classified as Stage I.

### 3.3. Maternal and Fetal Outcomes

Immune-related adverse events were documented in three pregnancies (3/21, 14.2%), including maternal hypophysitis, hepatitis, rash, and vitiligo. Serious pregnancy-related complications included fetal growth restriction (6/21, 28.5%), HELLP syndrome (2/21, 9.5%), maternal cholestasis (1/21, 4.7%), spontaneous abortion (1/21, 4.7%), stillbirth (1/23, 4.3%), and placental insufficiency with fetal heart abnormalities (1/21, 4.7%). No maternal deaths were reported.

**Figure 1:**
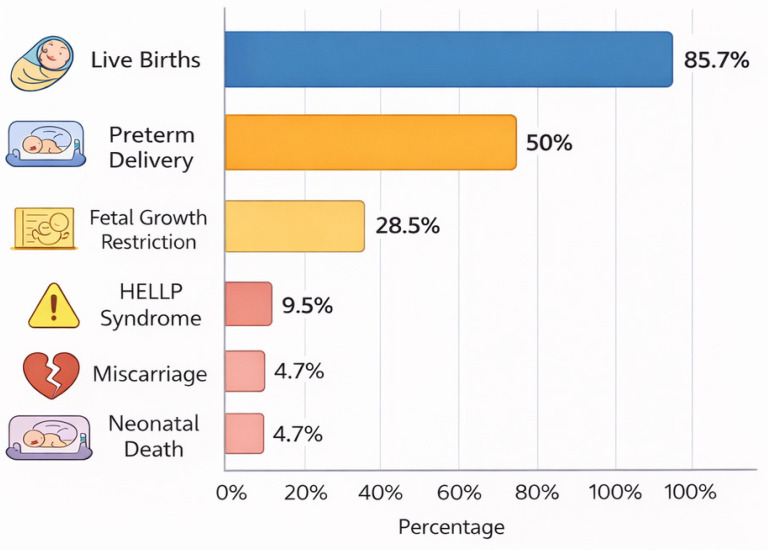
Summary of maternal and fetal outcomes following immune checkpoint inhibitor (ICI) exposure during pregnancy based on published cases.

Live births were reported in 85.7% (18/21) of ICI-exposed pregnancies. Of the 14 cases with documented gestational age at delivery, 7 (50%) were delivered preterm (range 26–33 weeks), typically due to planned early induction or cesarean section for maternal or obstetric indications, rather than spontaneous preterm labor. The remaining seven cases (50%, 7/14) were delivered at term [10]. A summary of reported maternal and fetal outcomes is presented in Figure 1.

Complications associated with prematurity were reported in five infants (5/23, 21.7%), including respiratory distress syndrome (RDS), intraventricular hemorrhage (IVH), retinopathy of prematurity (ROP), fetal distress, motor developmental delay, and stroke [9]. One infant presented with SCID-like features requiring ventilatory support. Additional outcomes included one neonatal death (4.7%) due to extreme prematurity and respiratory failure. NICU admissions were frequent among preterm infants, particularly those with fetal growth restriction or respiratory compromise (rows 2, 5 and 6 in the table). Congenital anomalies were observed in four infants (4/23, 17.4%), including one case of congenital hypothyroidism, one case of transient congenital hypothyroidism, and two cases of hand malformation.

Of the seven term infants, six were born healthy with normal growth and neurodevelopment. One infant, exposed to pembrolizumab from 16 weeks’ gestation through the late third trimester, developed severe immune-mediated gastroenterocolitis at four months of age, presenting with intractable diarrhea, failure to thrive, and hypoalbuminemia [23]. Extensive immunologic and histopathologic evaluation supported a diagnosis of pembrolizumab-induced immune toxicity. The infant required prolonged immunosuppressive therapy, including corticosteroids, infliximab, and azathioprine. His symptoms resolved and, by age two, he showed normal growth and intestinal mucosal recovery [23]. This case highlights the potential for delayed, transplacental ICI effects on neonatal immunity. Long-term pediatric follow-up data remain limited. A detailed overview of all published cases, including maternal cancer type, ICI regimen, and pregnancy outcome, is summarized in Table 1.

## 4. Case Series

### 4.1. Case 1

A 22-year-old non-Hispanic white woman presented in mid-2021 with a rapidly enlarging pigmented lesion on her posterior scalp, initially noticed as a raised bump. She also reported a palpable left neck mass. Dermatologic evaluation and shave biopsy of the scalp lesion revealed invasive nodular melanoma with a Breslow thickness of at least 4.0 mm, Clark level IV, ulceration, and brisk tumor-infiltrating lymphocytes. Fine needle aspiration of a left supraclavicular lymph node confirmed stage IIIC metastatic melanoma.

She began neoadjuvant pembrolizumab 200 mg IV every three weeks on 29 August 2021. On 21 October 2021, she underwent left neck dissection, which demonstrated metastatic melanoma in one of 27 lymph nodes. Histology showed therapy-induced necrosis, fibrosis, and melanosis, suggesting a partial response (50% viable tumor). Pembrolizumab was resumed postoperatively, for a total of 10 intended cycles. Her treatment course was complicated by the development of thyroiditis, a known immune-related adverse event.

At the time of ICI initiation, the patient was counseled regarding contraceptive use. She declined resumption of her recently discontinued combined oral contraceptives and adopted physical barrier contraception. She conceived during treatment, with estimated conception date of 13 April 2022, while receiving pembrolizumab. Immunotherapy was discontinued upon pregnancy confirmation; her last cycle (10th cycle) was administered on 18 April 2022.

The pregnancy course was unremarkable. She remained clinically well, reported no residual treatment toxicity, and was monitored closely throughout gestation. She delivered a healthy infant at 39.1 weeks of gestation. Placental pathology was unremarkable, and no neonatal complications were reported. At last follow-up in January 2024, both patient and child were doing well.

Reproductive hormone assessments pre- and post-treatment showed preserved ovarian reserve. Her pre-treatment AMH was 2.3 ng/mL and 3.1 ng/mL post-treatment. FSH and estradiol levels following ICI treatment were 2.6 mIU/mL and 95 pg/mL, respectively, consistent with intact hypothalamic–pituitary–ovarian axis function.

### 4.2. Case 2

A 37-year-old Hispanic woman presented in May 2019 with a palpable lymph node in the left lateral posterior neck. Biopsy revealed metastatic melanoma (7.1 × 6.0 mm) of unknown primary, involving a single left cervical lymph node. Molecular testing identified a BRAF V600E mutation. At diagnosis, she was two months postpartum with excellent performance status. She began neoadjuvant nivolumab (480 mg IV every four weeks) in July 2019 with curative intent, completing 10 of 12 planned cycles by March 2020. Her only treatment-related reported complaint was transient blurry vision shortly after the first dose, which resolved without sequelae. The patient was counseled regarding contraception prior to initiation of immunotherapy. Contraception during therapy consisted of condom use; the patient denied amenorrhea and maintained regular menses throughout treatment. The patient conceived in mid-March 2020, approximately eight months after initiating immunotherapy in July 2019. At the time of pregnancy confirmation, she was estimated to be about two weeks into gestation and had completed 10 of 12 planned cycles of nivolumab. Immunotherapy was discontinued upon confirmation. The pregnancy was later complicated by a spontaneous abortion at 13 weeks of gestation. She reported no immune-related toxicity at the time, and no alternate cause for pregnancy loss was identified. ICI therapy was not resumed. Following this pregnancy loss, she subsequently conceived again and delivered a healthy child at term without complications.

The patient’s post-treatment course has remained stable, with no evidence of disease recurrence. As of January 2024, she continues under routine surveillance.

## 5. Discussion

**Figure 2:**
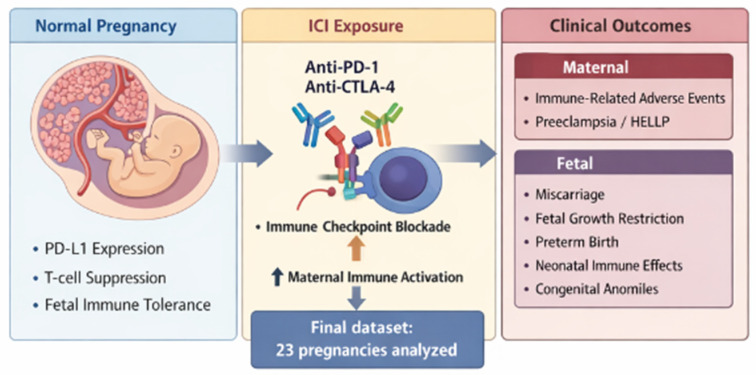
Proposed mechanisms and clinical implications of immune checkpoint inhibitor (ICI) exposure during pregnancy.

We report two pregnancies that were conceived inadvertently while patients were receiving ICI therapy for malignancy. The potential mechanisms underlying adverse maternal and fetal outcomes following ICI exposure are illustrated in Figure 2. Several mechanisms have been proposed for potential adverse obstetric outcomes with ICI exposure. Pregnancy is an immune-tolerant environment, partly mediated by immune checkpoints. PD-1/PD-L1 signaling helps the placenta evade maternal immune rejection, with PD-L1 abundantly expressed on trophoblasts to suppress maternal T-cell activity against fetal antigens [6]. Blocking this pathway with therapeutic antibodies could, in theory, disrupt tolerance. Animal studies support this premise, as administration of anti-PD-L1 antibodies to pregnant mice leads to increased fetal resorption and smaller litter sizes [25]. CTLA-4, another checkpoint upregulated on regulatory T-cells, also contributes to fetomaternal tolerance and its inhibition (e.g., with ipilimumab) in primate models resulting in increased miscarriage and perinatal mortality [26]. These data support concerns that ICI exposure, particularly in early pregnancy, may induce miscarriage or fetal demise. In humans, however, ICI exposure early in pregnancy did not universally result in immediate pregnancy loss, suggesting that outcomes may depend on timing, extent of blockade and other factors.

This adds to the growing body of evidence needed to understand the implications of ICI therapy in pregnancy. In our series, maternal and fetal outcomes were consistent with prior reports. Similar to prior reports in the literature [15], an uncomplicated pregnancy was achieved despite ICI therapy at time of conception although a second pregnancy was complicated by a late first-trimester pregnancy loss. This aligns with existing variability in reported outcomes, including second-trimester losses [16]. While live births have been documented despite ICI exposure, our review reinforces the need for cautious interpretation and close monitoring due to potential immune-related and fetal complications.

A pharmacovigilance study of VigiBase, the global adverse drug reactions database of the World Health Organization investigated 103 safety reports of ICI use during the peri-pregnancy period [4]. Among these reports, 56 (54%) described at least one pregnancy-related complication, with the majority involving fetal or neonatal outcomes (65%) and remaining cases reporting maternal complications [4]. The most commonly reported fetal outcomes were prematurity (32.1%), spontaneous abortion (21.4%) and fetal growth restriction (10.7%), with additional reports of hypoxia, stillbirth, and congenital anomalies including hypospadias and congenital hypothyroidism [4]. Despite these findings, the VigiBase analysis concluded that ICI exposure was not associated with a statistically significant increase in maternal complications such as preeclampsia, HELLP syndrome, or placental infarction compared with traditional antineoplastic agents, nor with an increased risk for spontaneous abortion among women aged 20–44 years (HR; 1.32 95% CI: 0.65–2.67) [4]. In contrast, our findings demonstrate a higher burden of obstetric and neonatal morbidity, including higher rates of fetal growth restriction (28.5%), HELLP syndrome (9.5%), and preterm delivery (50%). In addition to the congenital anomalies reported by VigiBase, our findings reported two cases of hand malformation. These differences likely reflect limitations inherent to spontaneous reporting databases and the use of comparator cancer therapies already associated with adverse pregnancy outcomes, which may attenuate detection of ICI-specific risk. Taken together, our findings suggest that ICI exposure during pregnancy may be associated with a higher burden of adverse obstetric and neonatal outcomes than suggested by pharmacovigilance data alone.

As pregnancy advances, another concern is transplacental transfer of the IgG monoclonal antibodies. Human IgG crosses the placenta, particularly after the first trimester, via Fc receptors. Notably, the IgG subclasses of the approved ICIs (e.g., IgG4 for nivolumab/pembrolizumab and IgG1 for ipilimumab) are expected to reach fetal circulation. This raises the possibility of direct drug effects on the fetus or neonate. A case of neonatal immune-mediated colitis after second-trimester pembrolizumab exposure illustrates this potential [23].

The prolonged half-life of ICIs (typically ~2–4 weeks) allows drug levels to persist in the maternal circulation for months [27]. Even if therapy is discontinued upon recognition of pregnancy, residual drug may expose the embryo/fetus during early organogenesis, complicating risk mitigation. There are no evidence-based protocols for timing of discontinuation in pregnancy. Risk likely varies by exposure period: first-trimester exposure raises concerns about teratogenesis and miscarriage, whereas third-trimester exposure may have greater newborn impact (e.g., immune-mediated neonatal conditions or complications from preterm delivery) [10]. Notably, many reported third-trimester ICI exposures are associated with preterm birth, often due to planned early delivery to limit fetal drug exposure. Some clinicians advocate delivery at 34–37 weeks to avoid ICI dosing in the final 6–8 weeks of pregnancy. This allows drug levels to wane prior to birth and reduce potential neonatal immune effects [10]. This approach is extrapolated from practices with other monoclonal antibodies in pregnancy and remains under discussion.

### 5.1. Clinical Management and Counseling

Given the profound uncertainties, a cautious and individualized approach is essential when managing pregnancy during ICI therapy. Multidisciplinary care—including oncology, maternal-fetal medicine, neonatology, and immunology—is essential. Patients should be counseled that, while healthy births have been reported, serious potential complications remain, including preeclampsia, fetal growth restriction, preterm birth, and rarely fetal or neonatal harm. The decision to continue or hold immunotherapy during pregnancy must balance maternal cancer prognosis and treatment alternatives. All patients of childbearing potential starting ICIs should be informed of these risks upfront and advised regarding effective contraception during therapy. If a patient conceives during therapy, thorough counseling about known risks and potential long-term effects is necessary.

For patients continuing pregnancy with ICI exposure, enhanced monitoring is recommended. This may include frequent ultrasound assessments for fetal growth and placental function, given the association with growth restriction and placental insufficiency [9,10,18]. Vigilance for hypertensive disorders of pregnancy is warranted, as immune activation could contribute to preeclampsia. Some experts suggest monitoring maternal immune status or drug levels, though no standardized approach exists [4]. Oncologists should also monitor for immune-related toxicities that could impact pregnancy, such as endocrine dysfunction [12]. Delivery planning should involve a multidisciplinary team, including oncology, maternal fetal medicine and neonatology prepared for potential immune or prematurity-related complications.

### 5.2. Knowledge Gaps and Research Needs

We are still in the early stages of understanding checkpoint inhibitor effects in pregnancy. Most available data come from case reports subject to publication bias (often emphasizing unusual or complicated outcomes). There is a pressing need for more systematic data collection. Prospective pregnancy registries for patients on ICIs would capture outcomes more reliably and facilitate long-term follow-up of exposed offspring. Additionally, basic research regarding ICI placental interactions and the fetal immune system would elucidate potential mechanisms for autoimmune complications (e.g., examining placental tissue from exposed pregnancies for immune infiltration or checkpoint expression alterations). Collaborative reporting of cases and international data-sharing will be critical to improve guidance.

## 6. Conclusions

The intersection of cancer immunotherapy and pregnancy is an emerging clinical reality. As ICIs become standard-of-care in younger patients, oncologists and obstetricians will increasingly encounter pregnant patients exposed to PD-1, PD-L1, or CTLA-4 inhibitors. The limited evidence suggests that favorable pregnancies are possible. However, significant risks remain—particularly prematurity, preeclampsia, fetal growth restriction and the potential for adverse neonatal immune dysregulation [10]. Patients utilizing ICIs should be counseled to avoid conception during therapy. If pregnancy does occur, a tailored plan should be developed, often favoring ICI discontinuation if clinically feasible. When immunotherapy must continue, intensive monitoring for maternal and fetal complications is advised, including anticipation of preterm delivery.

In conclusion, pregnancy during ICI therapy presents a challenging scenario at the frontiers of oncology and maternal-fetal medicine. Although current data does not suggest a major teratogenic effect, the long-term consequences of in utero exposure are unknown, and safe thresholds of fetal drug exposure remain undefined. We advocate for the creation of pregnancy exposure registries and international reporting systems to accumulate data on maternal and offspring outcomes and inform future guidelines. Until then, managing ICI exposure in pregnancy will require individualized clinical judgment, vigilant care, and open communication with patients regarding the uncertainties.

## Figures and Tables

**Table 1:** Reported cases of immune checkpoint inhibitor (ICI) exposure during pregnancy (ordered by year of publication).

Author/Year	Maternal Age (Years)	Gravidity/Parity	Immunotherapy	Malignancy	Exposure Timing (GA Weeks)	On ICI at Conception	Gestational Age at Birth	Birthweight (g)	Mode of Delivery	Adverse Events—Maternal	Adverse Events—Fetal/Neonatal	ICI-Related Adverse Events	Fetal Outcome	Maternal Outcome/Follow-Up
Niemi et al., 2017 [14]	35	NR	Nivolumab ± Ipilimumab	Melanoma	24 + 3 weeks	No	24+5 weeks	NR	NR	Preterm delivery	RDS, SCID (suspected), ventilator support	NR	Severe prematurity complications	NR
Mehta et al., 2018 [15]	33	NR	Ipilimumab	Melanoma	Preconception–9 weeks	Yes	≥38 weeks	NR	NR	None	None	G1 diarrhea	Healthy at 2 years	PD during pregnancy; pembrolizumab postpartum
Menzer et al., 2018 [16]	34	NR	Nivolumab + Ipilimumab	Melanoma	21–24 weeks	No	24+2 weeks	590	NR	Preterm birth (likely iatrogenic)	RDS, IVH, ROP, motor delay	None	Neonatal death (day 1)	NR
Burotto et al., 2018 [17]	34	NR	Nivolumab + Ipilimumab	Melanoma	9 weeks–2nd trimester	No	32 weeks	1640	NR	Placental insufficiency	None	Immune hepatitis (G3–G4)	Healthy infant	PD postpartum; PR to therapy
Xu et al., 2019 [18]	32	NR	Nivolumab + Ipilimumab	Melanoma	Preconception–7 + 6 weeks	Yes	33 weeks	1400	NR	IUGR	Congenital hypothyroidism	Multiple irAEs	NICU; normal development	CR postpartum
Bucheit et al., 2020 [19]	32	NR	Ipilimumab + Nivolumab	Melanoma	Since conception	Yes	32 weeks	1530	NR	Anemia, IUGR	NICU admission	NR	Healthy	Postpartum seizure
Bucheit et al., 2020 [19]	32	NR	Nivolumab + Ipilimumab	Melanoma	Preconception–32 weeks	Yes	32 weeks	Twin A: 1530; Twin B: 1700	NR	IUGR (twins)	NICU stays	None	Healthy	Seizure postpartum
Polnaszek et al., 2021 [20]	23	NR	Pembrolizumab	PSTT	Up to 6 weeks	Yes	39+4 weeks	NR	NR	None	None	NR	Healthy	No recurrence
Haiduk & Ziemer, 2021 [21]	39	NR	Nivolumab	Uveal melanoma	Preconception–6 weeks	Yes	30 weeks	1055, 950	NR	HELLP syndrome, IUGR	Hand malformation (1 twin)	None	Mixed outcomes	CR postpartum
Hutson et al., 2022 [22]	NR	NR	Nivolumab	Hodgkin lymphoma	1st trimester (residual)	Yes	Term	3200	NR	None	None	NR	Healthy	Complete remission
Salehi et al., 2022 [12]	34	NR	Nivolumab + Ipilimumab	Melanoma	27 weeks	NR	37 weeks	3200	NR	NR	None	NR	Healthy	Stable disease
Salehi et al., 2022 [12]	33	NR	Nivolumab	RCC	8 weeks	NR	38 weeks	3300	NR	NR	None	NR	Healthy	Partial response
Baarslag et al., 2023 [23]	26	NR	Pembrolizumab	Melanoma	16–37 weeks	No	37 weeks	3300	NR	Cholestasis	Immune-mediated enteritis	None	Recovered	Stable
Gougis et al., 2024 (multiple cases) [9]	20s–30s	NR	Various ICIs	Multiple	NR	NR	NR	NR	NR	HELLP, IUGR, miscarriage	Neonatal death, malformations	NR	Mixed outcomes	NR
Mastricci et al., 2025 [24]	NR	NR	Pembrolizumab	Hodgkin lymphoma	5–24 weeks	Yes	37.5 weeks	2650	NR	NR	None	NR	Healthy	Good outcome

Abbreviations: RDS, respiratory distress syndrome; IVH, intraventricular hemorrhage; ROP, retinopathy of prematurity; NICU, neonatal intensive care unit; SCID, severe combined immunodeficiency; IUGR, intrauterine growth restriction; HELLP, hemolysis, elevated liver enzymes, low platelets; PD, progressive disease; PR, partial response; CR, complete response; NR, not reported; GA, gestational age.

## Data Availability

The data presented in this study are not publicly available due to patient privacy and institutional confidentiality restrictions. De-identified data may be available from the corresponding author upon reasonable request and with permission from The University of Texas MD Anderson Cancer Center.

## Supplementary Materials

The following supporting information can be downloaded at: https://www.mdpi.com/article/10.3390/curroncol33060318/s1, Table S1: Summary of Reported Maternal Immune Checkpoint Inhibitor Exposure During Pregnancy and Outcomes [9,12,14,15,16,17,18,19,20,21,22,23,24].
