# Supplementary material for: Pregnancy Outcomes After in Utero Exposure to Immune Checkpoint Inhibitors
Source: Curr Oncol. 2026 May 28;33(6):318. doi: 10.3390/curroncol33060318 (PMC13298528; doi:10.3390/curroncol33060318)
Supplement: Supplementary file 1 [file curroncol-33-00318-s001.zip › curroncol-4205811-supplementary.pdf]

Supplementary Table

Author/Date	Stage at Pregnancy	Age at Pregnancy	Cumulative Number of weeks of exposure during pregnancy	Total Dose Administered	PFS (months)	OS (months)
Bucheit et al./ 2020	Metastatic	32	Continued throughout pregnancy	NR	NED	NR
Mehta et al., 2018	IV	33	Before pregnancy–9th GA week	NR	7	>30
Menzer et al., 2018	IV	34	21st–24th GA week	3 doses Ipi, 4 doses Nivo (est.)	1	1
Bucheit et al., 2020	IV	32	Before pregnancy–32nd GA week	NR	>12	>12
Xu et al., 2019	IV	32	Before pregnancy–7+6 GA week	NR	>27.25	>27.25
Burotto et al., 2018	IV	34	9th GA week–2nd trimester	Ongoing until 32nd week; not specified	6	>11
Niemi et al., 2017	IV	35	24+3 GA week	2 doses Nivo (est.)	>0.07	>0.07
Gougis et al., 2024	III	30s	NR	2 doses Nivo, 1 dose Ipi	NR	NR
Gougis et al., 2024	NR	NR	NR	1 dose Ipi, 1 dose Nivo	NR	NR
Gougis et al., 2024	NR	30s	NR	NR	NR	NR
Gougis et al., 2024	NR	30s	NR	NR	NR	NR
Gougis et al., 2024	NR	NR	NR	NR	NR	NR
Gougis et al., 2024	NR	30s	NR	NR	NR	NR
Gougis et al., 2024	NR	20s	NR	NR	NR	NR
Salehi et al., 2022	Metastatic	34	NR	NR	NR	NR
Salehi et al., 2022	Metastatic	33	NR	NR	NR	NR
Haiduk & Ziemer, 2021	Metastatic	39	Before pregnancy to 6 weeks GA	10 doses	>19.25	>19.25
Mastricci et al., 2025	NR	NR	1st and 2nd trimester (5 cycles)	11 cycles	NR	NR
Hutson et al., 2022	Remission	NR	1st trimester (1 dose indirectly exposed)	1 dose	NR	NR
Polnaszek et al., 2021	I	23	Up to 6 weeks GA	3 cycles pembrolizumab 200 mg	NR	NR
Baarslag et al., 2023	IIIB	26	16–37 weeks GA (~4 doses)	~4 cycles (400 mg q6 weeks)	NR	NR
